# Prognostic Factors of 30-Day In-Hospital Mortality in Critically Ill Patients Receiving Continuous Renal Replacement Therapy

**DOI:** 10.1097/jnr.0000000000000734

**Published:** 2026-03-11

**Authors:** Hyeon-Ju LEE, Taehee KIM, Heeyoung LEE, Youngeon LEE, Jinseon HEO, Youn-Jung SON

**Affiliations:** 1 Department of Nursing, Tongmyong University, Busan, South Korea; 2 Department of Internal Medicine, Inje University College of Medicine, Busan, South Korea; 3 Department of Nursing, Inje University Busan Paik Hospital, Busan, South Korea; 4 Emergency Intensive Care Unit, Department of Nursing, Chung-Ang University Hospital, Seoul, South Korea; 5 College of Nursing, Kosin University, Busan, South Korea; 6 Red Cross College of Nursing, Chung-Ang University, Seoul, South Korea

**Keywords:** continuous renal replacement therapy, hospital mortality, critical care nursing, retrospective study, risk factors

## Abstract

**Background:**

Continuous renal replacement therapy (CRRT) is a form of dialysis that effectively replicates the excretory function of the kidneys in critically ill patients suffering from acute kidney injury. The number of patients receiving CRRT in critical care settings is increasing globally. Although these patients are at greater risk of mortality after commencing CRRT, the comprehensive risk factors for in-hospital mortality in this population remain uncertain.

**Purpose:**

The prognostic factors for 30-day in-hospital mortality in patients receiving CRRT are investigated in this study.

**Methods:**

This retrospective observational study was performed at a tertiary care university hospital between January 2018 and December 2020. Data from a total of 613 patients requiring CRRT were included. Pre-, intra-, and post-CRRT data were extracted from electronic medical records, and patients were grouped based on 30-day in-hospital mortality into survivor (*n* = 300) and nonsurvivor (*n* = 313) groups.

**Results:**

The mortality rate within 30 days of hospitalization was 51.1%. The median survival time calculated using the Kaplan–Meier method was 9 days. The results of multivariate Cox regression analysis revealed hepatic failure as a comorbidity (adjusted hazard ratio [HR] = 2.75, 95% confidence interval [CI] = [1.15, 6.58]), post–continuous renal replacement therapy data including Glasgow Coma Score (adjusted HR = 0.82, 95% CI = [0.72, 0.94]), Sequential Organ Failure Assessment Score (adjusted HR = 1.16, 95% CI = [1.02, 1.32]), and sodium level (adjusted HR = 1.05, 95% CI =[1.01, 1.10]) to be linked to higher in 30-day in-hospital mortality for patients receiving CRRT.

**Conclusions/Implications for Practice:**

Based on the findings, early detection and management of changes in illness severity, consciousness levels, and sodium levels during CRRT should be emphasized in ICU care settings. The authors hope the findings contribute to the design and application of transitional care plans for patients undergoing CRRT.

## Introduction

Continuous renal replacement therapy (CRRT) is a special type of dialysis given to unstable patients in the intensive care unit (ICU) whose bodies cannot tolerate regular hemodialysis (Bourbonnais et al., 2020). This modality is attractive because net fluid removal can be extended over 24 hours instead of the usual 4 hours for hemodialysis, thereby reducing the risk of hypotension (Tandukar & Palevsky, 2019). CRRT is the preferred option in critically ill patients who suffer from acute kidney injury (AKI), with or without baseline chronic renal impairment (Ahmed et al., 2019). CRRT is also utilized in cases of multiorgan failure and sepsis (Tandukar & Palevsky, 2019).

CRRT is performed to provide kidney supportive care in ~10%–40% of critically ill patients (Ahmed et al., 2019; Tiglis et al., 2022). Despite the efforts to treat patients in need of CRRT, mortality rates among this population remain high (Tiglis et al., 2022). According to a recent review (H.-J. Lee & Son, 2020), in-hospital mortality rates vary between 38.6% and 62.4% for patients receiving CRRT, while the rate for patients with AKI requiring CRRT in the ICU ranges from 50% to 75% (Rewa et al., 2023). High mortality rates after CRRT may increase length of hospital stays, medical service durations, and health care costs (Järvisalo et al., 2022). The findings of a systematic review highlighted that investigating the 30-day in-hospital mortality may be beneficial in providing timely renal care after commencing CRRT (Xia et al., 2021). However, few studies have been published regarding the risk factors of 30-day in-hospital mortality in patients on CRRT in ICU settings (Priyamvada et al., 2018). Thus, determining the factors contributing to mortality risk in patients undergoing CRRT is vital to reducing avoidable deaths and developing transitional care plans from the ICU to home.

Based on the literature, older age, a high number of comorbidities, and low body mass index (BMI) have been linked to higher mortality risk in patients on CRRT (H.-J.Lee & Son, 2020; Rewa et al., 2023). In addition, unstable hemodynamic factors such as lower systolic and diastolic blood pressure values, extracorporeal membrane oxygenation use, and higher severity of illness scores have all been associated with a greater risk of in-hospital mortality in patients on CRRT (Jeon et al., 2021; Jiang et al., 2022; Peters et al., 2023). However, conflicting and inconsistent results have been reported. Moreover, existing evidence on the risk factors for mortality has focused primarily on a single time period, for example, pre- or post-CRRT (Jeon et al., 2021; Zhou et al., 2023). Thus, critical care teams should consider integrative risk assessment approaches to ensure patient safety during CRRT (Uusalo et al., 2021). ICU nurses are frontline health care professionals responsible for regularly monitoring and managing patients undergoing CRRT (Bourbonnais et al., 2020). In a prior study, nurses were shown to have played a key role in decreasing in-hospital mortality by 14% (Bourbonnais et al., 2020). In this respect, ICU nurses should keep in mind the significance of identifying risk factors related to in-hospital mortality among their patients on CRRT.

Recently, CRRT utilization has increased in many countries, including Korea, based on its benefits in the treatment of AKI and other critical illnesses (Zhou et al., 2023). However, clear evidence regarding the factors associated with the increased risk of mortality in patients on CRRT is not available (S. Park et al., 2018). Furthermore, a recent meta-analysis reported differences in CRRT outcomes due to heterogeneity among the regions in which the studies were conducted (Chander et al., 2024). Therefore, the objective of this study was to investigate the prevalence and prognostic factors of 30-day in-hospital mortality among Korean patients on CRRT. The findings are expected to contribute to the global response by providing evidence related to short-term mortality in CRRT patients.

## Methods

### Study Design and Settings

Data for this retrospective observational study were obtained from the electronic medical records of all patients admitted to the medical and surgical ICU of a single tertiary care university hospital with 842 beds in South Korea from January 2018 to December 2020.

### Samples

**Figure 1:**
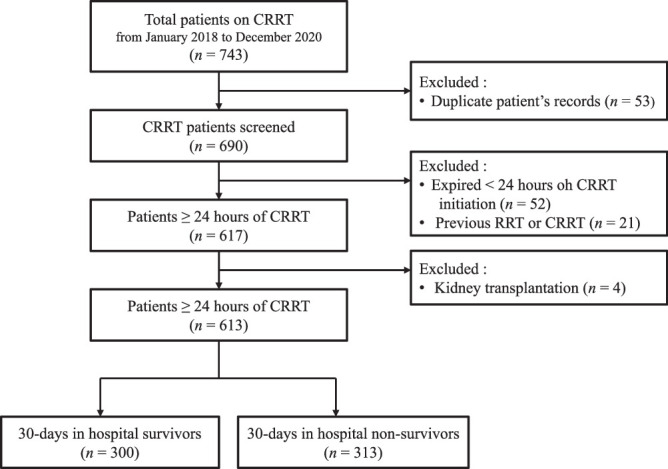
*Flow Diagram of the Study* *Note.* CRRT = continuous renal replacement therapy.

Hospital electronic medical records from January 2018 to December 2020 were analyzed, and 743 ICU patients aged ≥18 years on continuous venovenous hemodiafiltration were screened. Continuous venovenous hemodiafiltration provides both diffusive and convective solute clearance (Peters et al., 2023). After excluding 53 duplicate records, 690 medical records were included in the initial sample. Records of patients with any of the following criteria were excluded: (1) died within the first 24 hours of CRRT (*n* = 52), (2) previous recipient of RRT or CRRT (*n* = 21), and (3) recipient of kidney transplantation (*n* = 4). The final sample included the medical records of 613 patients (Figure 1).

A sample size calculation using G*power 3.1 was conducted (Faul et al., 2009). Based on previous studies (H.-J. Lee & Son, 2020; Yang et al., 2016), a minimum sample size of 620 is required to identify a predictor variable with an *OR* of 1.3 in a population with 80% power and a two-sided α level of .05. Thus, the 613-patient sample in this study was somewhat insufficient.

### Measures

#### Patient general characteristics at baseline

Data on patient age, gender, smoking status, alcohol consumption, and BMI (kg/m^2^) were collected. Comorbidities were categorized into the following categories: diabetes mellitus, hypertension, cerebrovascular disease, heart failure, respiratory failure, hepatic failure, and cancer.

#### Pre-CRRT patient characteristics

Pre-CRRT characteristics data were collected before CRRT initiation based on a literature review (Peters et al., 2023; Tiglis et al., 2022). Cardiopulmonary resuscitation (CPR), mechanical ventilation use, and the Glasgow Coma Scale (GCS) were used to evaluate the patient’s level of consciousness (Bodien et al., 2021). Sequential Organ Failure Assessment (SOFA) score, mean arterial pressure (MAP), and presence of oliguria information, as well as laboratory data such as creatinine, sodium, potassium, total bilirubin, hemoglobin, platelets, and lactic acid levels, were collected. The SOFA score is widely used for the prognostication and assessment of multiple organ dysfunctions in critically ill patients (Bahtouee et al., 2019). This score is the sum of 6 organ dysfunctions (respiratory, cardiovascular, hepatic, coagulation, renal, and neurological systems). The SOFA score was calculated at hospital admission and every 24 hours until ICU discharge or death for every included patient. SOFA total scores range from 0 (*normal organ function*) to 4 (*worst organ function*), with higher scores reflecting higher organ failure progression. The mean SOFA was calculated as the average of daily SOFA scores for each patient over their ICU stay.

In this study, the average MAP value was calculated using the MAP values taken 24 hours before CRRT initiation.

#### Intra-CRRT patient characteristics

The intra-CRRT characteristics included data related to the application of CRRT. Data included the site of vascular access, use of anticoagulants, and average prescribed dose (mL/kg/h). The average prescribed dose for each patient was derived from the prescribed dose based on the duration of CRRT (H.-J. Lee & Son, 2020; K.Y. Lee et al., 2022).

#### Post-CRRT patient characteristics

Post-CRRT characteristics included data from the day of CRRT device implantation to in-hospital survival within 30 days. Based on a literature review (Ahmed et al., 2019; Chander et al., 2024; Järvisalo, et al., 2022), data considered included use of mechanical ventilation, GCS and SOFA scores, average MAP, duration of CRRT (in days), and laboratory data such as creatinine (mg/dL), sodium (mg/dL), potassium (mg/dL), total bilirubin (mg/dL), hemoglobin (g/dL), platelets (10^3^/μL), and lactic acid (mg/dL) levels. In patients who were sedated, the GCS score was evaluated by the ICU nurse during the sedation break. Average MAP values for the survivor and nonsurvivor groups were calculated as the average value from CRRT initiation until, respectively, just before stopping CRRT and just before death.

#### 30-day in-hospital mortality

The 30-day in-hospital mortality was used as the primary outcome. Death was defined as all-cause in-hospital death during hospitalization within 30 days of CRRT initiation.

### Ethical Considerations and Data Collection

Institutional review board approval was obtained from Chung-Ang University (IRB No. 2020-12-046-002) before commencing this study. The patient records were collected retrospectively and closely reviewed by the researchers. Medical record reviews included hospital and ICU admissions, CRRT notes, nursing records from ICUs and general wards, medication administration records, laboratory data, and discharge records. Patient identification numbers were deleted for anonymization purposes during data collection.

### Data Analysis

All data analysis was conducted on SPSS version 27.0 (IBM Corp., Armonk, NY, USA). Continuous variables were summarized as mean with *SD*; categorical variables were reported as frequencies with percentages; and independent *t* tests, χ^2^ tests, or Fisher’s exact tests were used to confirm differences in baseline patient characteristics and pre-, intra-, and post-CRRT data between nonsurvivors and survivors.

The 30-day in-hospital survival probability was analyzed using Kaplan–Meier methods. Log-rank tests were utilized to compare 30-day in-hospital survival probability between groups, and univariate and multivariable Cox proportional hazards models were used to confirm predictors of 30-day in-hospital mortality in adult patients on CRRT. To determine predictors of 30-day in-hospital mortality, statistically significant variables in the univariable analysis (*p* < .05) were maintained in the multivariable Cox proportional hazard regression model.

## Results

### In-Hospital Mortality and Between-Group Comparison of Baseline Patient Characteristics

The 30-day in-hospital mortality rate in the entire sample was 52.1% (*n* = 313). As shown in Table 1, the mean age of the sample was 69.35±13.19 years and the proportion of male patients was 54.6% (*n* = 335). Significant differences between the survivor and nonsurvivor groups were found in terms of BMI (*t* = 3.00, *p* = .003), comorbid diabetes mellitus (χ^2^ = 24.42, *p* < .001), hypertension (χ^2^ = 9.30, *p* = .002), hepatic failure (χ^2^ = 6.96, *p* = .008), and cancer (χ^2^ = 16.61, *p* < .001). No significant between-group differences were found for other baseline characteristics.

**Table 1:** Baseline Participant Characteristics (*N* = 613)

Characteristic	Total (*n* = 613)	Survivors (*n* = 300)	Nonsurvivors (*n* = 313)	*t* or χ^2^	*p*
	*n* (%)	*n* (%)	*n* (%)		
Age (years; *M* and *SD*)	69.35±13.19	69.26±13.15	69.43±13.25	−0.16	.870
Gender, male	335 (54.6)	157 (52.3)	178 (56.9)	1.27	.259
Smoking, yes	115 (18.8)	58 (19.3)	57 (18.2)	0.13	.722
Alcohol consumption, yes	135 (22.0)	70 (23.3)	65 (20.8)	0.59	.443
Body mass index (kg/m^2^; *M* and *SD*)	23.31±4.50	23.88±4.53	22.72±4.40	3.00	.003
Comorbidities ^a^					
Diabetes mellitus, yes	269 (43.9)	162 (54.0)	107 (34.2)	24.42	<.001
Hypertension, yes	317 (51.7)	174 (58.0)	143 (45.7)	9.30	.002
Cerebrovascular disease, yes	147 (24.0)	72 (24.0)	75 (24.0)	0.01	.991
Heart failure, yes	94 (15.3)	46 (15.3)	48 (15.3)	0.01	.999
Respiratory failure, yes	73 (11.9)	34 (11.3)	39 (12.5)	0.19	.667
Hepatic failure, yes	56 (9.1)	18(6.0)	32(12.1)	6.96	.008
Cancer	130 (21.2)	43 (14.3)	87 (27.8)	16.61	<.001

*Note.* CRRT = continuous renal replacement therapy.

^a^ Multiple response.

### Between-Group Differences in Pre-CRRT Variables

As shown in Table 2, significant between-group differences were found in use of mechanical ventilation (χ^2^ = 44.17, *p* < .001), GCS score (*t* = 7.65, *p* < .001), SOFA score (*t* = −9.05, *p* < .001), average MAP (*t* = 4.45, *p* < .001), creatinine level (*t* = 6.43, *p* < .001), sodium level (*t* = −2.61, *p* = .009), potassium level (*t* = 2.48, *p* =0.013), total bilirubin level (*t* = −4.72, *p* < .001), platelet level (*t* = 4.44, *p* < .001), and lactic acid level (*t* = −5.22, *p* < .001) before CRRT initiation. No significant between-group differences were found for other pre-CRRT characteristics.

**Table 2:** Between-Group Differences in Pre-CRRT and Intra-CRRT Characteristics (*N* = 613)

Characteristic	Total (*n* = 613)	Survivors (*n* = 300)	Nonsurvivors (*n* = 313)	*t* or χ^2^	*p*
	*n* (%) or Mean± *SD*	*n* (%) or Mean ± *SD*	*n* (%) or Mean ± *SD*		
**Pre-CRRT characteristics**					
CPR, yes	64 (10.4)	24 (8.0)	40 (12.8)	3.74	.053
Mechanical ventilation, yes	378 (61.7)	145 (48.3)	233 (74.4)	44.17	<.001
Glasgow Coma Scale	7.29±5.14	8.84±5.34	5.80±4.47	7.65	<.001
SOFA score	9.81±3.26	8.76±2.96	10.91±3.16	-9.05	<.001
Average MAP (mm Hg)	81.94±15.76	84.81±16.97	79.20±13.99	4.45	<.001
Oliguria before CRRT, yes	376 (61.3)	174 (46.3)	202 (53.7)	2.76	.097
Laboratory data					
Creatinine (mg/dL)	3.68±2.74	4.39±2.85	3.00±2.44	6.43	<.001
Sodium (mg/dL)	138.78±7.57	137.97±6.97	139.56±8.03	−2.61	.009
Potassium (mg/dL)	4.63±1.67	4.75±1.26	4.52±1.06	2.48	.013
Total bilirubin (mg/dL)	2.10±3.70	1.40±2.93	2.77±4.20	−4.72	<.001
Hemoglobin (g/dL)	9.85±2.23	9.87±2.06	9.83±2.38	0.22	.823
Platelets (10^3^/μL)	158.84±110.52	178.80±111.73	139.72±106.04	4.44	<.001
Lactic acid (mg/dL)	5.87±5.27	4.28±4.17	7.34±5.75	−5.22	<.001
**Intra-CRRT characteristics**					
Vascular access				3.68	.273 ^a^
Internal jugular vein	189 (30.8)	96 (32.0)	93 (29.7)		
Femoral vein	404 (65.9)	198 (66.0)	206 (65.8)		
Subclavian vein	2 (0.3)	1 (0.2)	1 (0.2)		
Others	18 (2.9)	5 (0.8)	13 (2.1)		
Anticoagulant, yes	344 (55.8)	172(57.0)	172(54.6)	0.35	.557
Average prescription dose (mL/kg/h)	44.46±11.01	42.93±9.05	45.92±12.45	−3.42	.001

*Note.* CRRT = continuous renal replacement therapy; CPR = cardiopulmonary resuscitation; SOFA = sequential organ failure assessment; MAP = mean arterial pressure.

^a^ Fisher’s exact test.

### Between-Group Differences in Intra-CRRT Variables

As shown in Table 2, the average prescription CRRT dose (*t* = −3.42, *p* = .001) was the only factor to reveal a statistically significant difference between survivors and nonsurvivors during the intra-CRRT period.

### Between-Group Differences in Post-CRRT Variables

As shown in Table 3, significant between-group differences were found in use of mechanical ventilation (χ^2^ = 124.96, *p* < .001), GCS (*t* = 18.07, *p* < .001), SOFA score (*t* = −23.93, *p* < .001), average MAP (*t* = 13.71, *p* < .001), creatinine level (*t* = 4.06, *p* < .001), sodium level (*t* = −6.08, *p* < .001), potassium level (*t* = −8.18, *p* < .001), total bilirubin level (*t* = −7.26, *p* < .001), platelet level (*t* = 10.60, *p* < .001), and lactic acid level (*t* = −13.91, *p* < .001) after CRRT implementation. No significant between-group differences were found for other post-CRRT characteristics.

**Table 3:** Between-Group Comparison of Post-CRRT Characteristics (*N* = 613)

Characteristic	Total (*n* = 613)	Survivors (*n* = 300)	Nonsurvivors (*n* = 313)	*t* or χ^2^	*p*
	Mean ± *SD*	Mean ± *SD*	Mean ± *SD*		
Mechanical ventilation, yes (*n*, %)	384 (62.6)	121 (4.3)	263 (84.0)	124.96	<.001
Glasgow Coma Scale	7.75±5.10	1.83 ±4.53	4.80±3.68	18.07	<.001
SOFA score	1.88±4.88	7.41±3.45	14.19±3.56	−23.93	<.001
Average MAP (mm Hg)	76.73±18.57	85.88±13.99	67.95±18.19	13.71	<.001
Laboratory data					
Creatinine (mg/dL)	1.25±0.92	1.40±0.94	1.10±0.87	4.06	<.001
Sodium (mg/dL)	138.44±5.61	137.08±3.33	139.75±6.96	−6.08	<.001
Potassium (mg/dL)	3.95±0.79	3.40±0.43	4.19±0.96	−8.18	<.001
Total bilirubin (mg/dL)	3.77±5.69	2.15±4.33	5.32±6.28	−7.26	<.001
Hemoglobin (g/dL)	9.13±1.52	9.11±1.25	9.15±1.75	−0.32	.751
Platelets (10^3^/μL)	101.33±76.53	132.29±81.15	71.67±58.08	1.60	<.001
Lactic acid (mg/dL)	7.87±8.06	3.02±5.24	12.18±7.68	−13.91	<.001
Duration of CRRT support (days)	6.47±5.18	6.28±4.62	6.65±5.67	−0.90	.369

*Note.* CRRT = continuous renal replacement therapy; SOFA = sequential organ failure assessment; MAP = mean arterial pressure.

### Predictors of 30-Day In-Hospital Mortality

**Figure 2:**
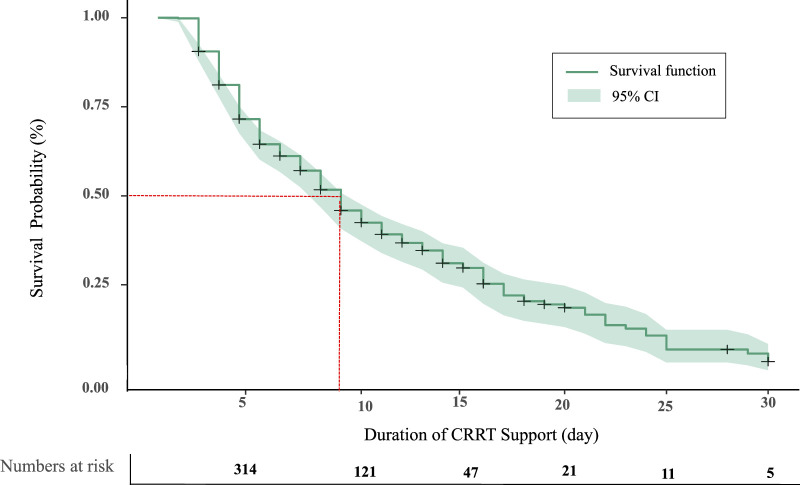
*Overall Survival Probability* *(* *%) of Patients on Continuous Renal Replacement Therapy* *Note.* CI = confidence interval; CRRT = continuous renal replacement therapy.

**Figure 3:**
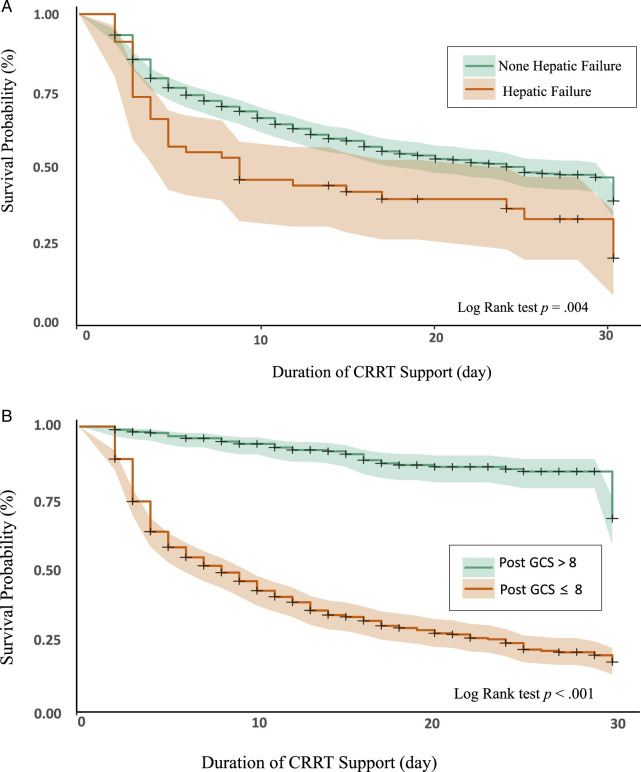
(A) *Probability of Survival With and Without Comorbid Hepatic Failure*. (B) *Probability of Survival Based on Post-CRRT GCS Score Range* *Note.* CRRT = continuous renal replacement therapy; GCS = Glasgow coma scale.

As shown in Figure 2, the results of the Kaplan–Meier analysis showed the 50th percentile of patients survived for 9 days after initiating CRRT, and that 30-day in-hospital mortality was significantly related to comorbid hepatic failure (*p* = .004) and post-CRRT GCS score (*p* < .001; Figure 3).

Notably, after controlling for confounding variables with *p* values < .05 in the univariate analysis, the multivariate Cox proportional hazards model revealed comorbid hepatic failure (adjusted HR = 2.75, 95% CI [1.15, 6.58], *p* = .023), post-CRRT GCS score (adjusted HR = 0.82, 95% CI [0.72, 0.94], *p* = .004), post-CRRT SOFA score (adjusted HR = 1.16, 95% CI [1.02,1.32], *p* = .026), and post-CRRT sodium level (adjusted HR = 1.05, 95% CI [1.01, 1.10], *p* = .026) to relate significantly to a high risk of 30-day in-hospital mortality (Table 4).

**Table 4:** Predictors of 30-Day In-Hospital Mortality (*N* = 613)

Predictor	Unadjusted HR (95% CI)	*p*	Adjusted HR (95% CI)	*p*
General characteristics				
Body mass index (kg/m^2^)	0.96 [0.93, 0.99]	.006	0.98 [0.92, 10.4]	.500
Diabetes mellitus, yes	0.58 [0.46, 0.73]	<.001	0.78 [0.47, 1.29]	.331
Hypertension, yes	0.78 [0.62, 0.97]	.026	0.97 [0.58, 1.63]	.927
Hepatic failure, yes	1.60 [1.14, 2.25]	.006	2.75 [1.15, 6.58]	.023
Cancer, yes	1.73 [0.35, 2.22]	<.001	1.06 [0.61, 1.82]	.847
Pre-CRRT characteristics				
Mechanical ventilation, yes	2.22 [1.72, 2.86]	<.001	1.69 [0.44, 6.46]	.443
Glasgow Coma Scale	0.92 [0.90, 0.94]	<.001	1.02 [0.90, 1.15]	.817
SOFA score	1.17 [1.13, 1.22]	<.001	0.91 [0.79, 1.04]	.174
Average mean arterial pressure (mm Hg)	0.96 [0.98, 0.99]	<.001	0.99 [0.97, 1.01]	.541
Creatinine (mg/dL)	0.85 [0.80, 0.90]	<.001	0.98 [0.84, 1.15]	.865
Sodium (mg/dL)	1.02 [1.01, 1.04]	.009	1.01 [0.98, 1.05]	.474
Potassium (mg/dL)	0.95 [0.86, 10.05]	.318	0.91 [0.71, 1.17]	.447
Total bilirubin (mg/dL)	1.04 [1.02, 1.06]	<.001	1.10 [1.00, 1.21]	.050
Platelets (10^3^/μL)	0.99 [0.98, 0.99]	<.001	1.00 [1.00, 1.01]	.834
Lactic acid (mg/dL)	1.07 [1.04, 1.98]	<.001	0.10 [0.94, 1.06]	.919
Intra-CRRT characteristics				
Average prescription dose (mL/kg/h)	1.02 [1.01, 1.03]	<.001	0.10 [0.98, 1.01]	.609
Post-CRRT characteristics				
Mechanical ventilation, yes	4.69 [3.46, 6.36]	<.001	0.48 [0.16, 1.49]	.205
Glasgow Coma Scale	0.80 [0.78, 0.83]	<.001	0.82 [0.72, 0.94]	.004
SOFA score	1.27 [1.23, 1.30]	<.001	1.16 [1.02, 1.32]	.026
Average mean arterial pressure (mm Hg)	0.96 [0.96, 0.97]	<.001	0.99 [0.98, 1.01]	.317
Creatinine (mg/dL)	0.83 [0.71, 0.97]	.016	1.03 [0.85, 1.26]	.758
Sodium (mg/dL)	1.07 [1.05, 1.09]	<.001	1.05 [1.01, 1.10]	.026
Potassium (mg/dL)	1.78 [1.58, 2.01]	<.001	1.13 [0.88, 1.46]	.346
Total bilirubin (mg/dL)	1.04 [1.02, 1.05]	<.001	0.95 [0.90, 1.00]	.050
Platelets (10^3^/μL)	0.99 [0.98, 0.99]	<.001	1.00 [0.99, 1.01]	.727
Lactic acid (mg/dL)	1.09 [1.08, 1.11]	<.001	1.02 [0.98, 1.06]	.430

*Note.* HR = hazard ratio; CI = confidence interval; CRRT = continuous renal replacement therapy; SOFA = sequential organ failure assessment.

## Discussion

Despite advancements in CRRT technology and its increased application among critically ill patients, in-hospital mortality rates among these patients remain high (Järvisalo et al., 2022). In this study, 30-day in-hospital mortality was shown to be high among ICU patients on CRRT (51.1%), which is consistent with studies reporting 30-day mortality rates of 66.2% (K. Y. Lee et al., 2022) and 51.0% (Peters et al., 2023), respectively. However, other prior research has shown significantly lower mortality rates (16.0%–33.3%; Kee et al., 2018; Prasad et al., 2016). The disparity between our findings and those of prior studies may relate to differences in the timing of the primary outcome measurement. In this study, the focus was on 30-day in-hospital mortality, as this time period is considered to offer an effective indicator of acute health care service quality (Xia et al., 2021). In particular, the findings of this study indicate mortality rates increase 9 days after CRRT unit initiation. Accordingly, ICU nurses should recognize the importance of early preventable risk factor screenings to reducing in-hospital mortality among patients on CRRT.

The main findings of this study show that comorbid hepatic failure and post-CRRT characteristics, such as poor GCS and SOFA scores and higher sodium levels, significantly increase 30-day in-hospital mortality risk in patients on CRRT. In this study, hepatic failure was identified as the strongest prognostic factor for 30-day in-hospital mortality. A previous study conducted in the United States found 70% of patients with acute hepatic failure had AKI and 30% required RRT (Tujios et al., 2015). In another study conducted in Europe, the reported incidence of AKI in patients with hepatic failure was 63.4% (Hadem et al., 2019). Liver cell death in hepatic failure can lead to renal tubular cell apoptosis in the kidneys (Sharma et al., 2024). In addition, Nishino et al. (2024) reported bilirubin levels, as an indicator of hepatic function, to be an independent prognostic factor for mortality among AKI patients with CRRT. Similarly, the univariate Cox regression model in this study found total bilirubin level to be higher in nonsurvivors than in survivors. Consequently, it is important to monitor changes in renal and liver functions continuously in patients with either hepatic failure or AKI, with more studies needed to identify causal relationships between the two conditions.

Second, better GCS scores during CRRT were linked to a decreased risk of 30-day in-hospital mortality. The GCS is often used to assess the level of consciousness in critically ill patients (Reith et al., 2016), and GCS scores of ≤8 are typically used to determine severe brain injury (Fitzgerald et al., 2022). In this study, the average GCS score was ~10 in the survivor group and 4 in the nonsurvivor group, which is in line with the between-group findings of previous studies (Ahmadi et al., 2023; Fitzgerald et al., 2022). Lower GCS scores may be considered an adverse outcome indicator of CRRT use, as rapid fluctuations in blood urea concentration caused by RRT may lead to changes in osmotic pressure, potentially leading to adverse outcomes such as cerebral edema and elevated intracranial pressure (Mistry, 2019).

According to a recent systematic review, patients with acute brain injury undergoing RRT due to AKI are more likely to experience neurological complications (Husain-Syed et al., 2023). That finding points to a need to promptly identify the causes of AKI and administer CRRT in a timely manner. However, that study relied on retrospective data only. Thus, large prospective studies are needed to determine the bidirectional relationship between GCS scores and the positive outcomes of CRRT in ICU patients. Moreover, studies designed to determine optimal GCS score cutoff points are needed to predict the likelihood of in-hospital mortality among ICU patients on CRRT.

Third, having a higher SOFA score during CRRT support was shown to be linked to a greater risk of 30-day in-hospital mortality in patients on CRRT. This finding supports previous findings that the SOFA score is significantly associated with mortality risk in critically ill patients with AKI on CRRT (Chang et al., 2014; Wang et al., 2020) but contradicts Schaffer et al. (2022), who reported that higher SOFA scores were not a risk factor for in-hospital mortality in patients on CRRT. Several studies have also reported that the Acute Physiology and Chronic Health Evaluation (APACHE) II and SOFA are reliable tools for predicting in-hospital mortality in ICU patients on CRRT (Gong et al., 2019; H. Park et al., 2023). Thus, further studies are required to identify which severity scoring systems are useful tools for assessing mortality risk in patients on CRRT and to develop kidney-specific severity scoring systems. Furthermore, multicenter, larger cohort studies are required to determine the optimal cutoff SOFA score for predicting high-risk patients on CRRT.

Finally, sodium levels during CRRT were identified in this study as a significant predictor of 30-day in-hospital mortality. The mean sodium levels in the nonsurvivor group were significantly higher than those in the survivor group during CRRT, even though the the level in both groups was maintained within the normal range of 135– 145 mEq/L. This finding is partly supported by a previous study, which related larger changes in serum sodium levels during CRRT to increased 90-day mortality risk among critically ill patients on CRRT (Petnak et al., 2022). However, in another previous study, sodium levels at the start of CRRT were identified as a significant predictor of mortality (Han et al., 2016). In this study, mean pre- and post-CRRT sodium levels in the nonsurvivor group were 139.56 and 139.75 mEq, respectively, which were slightly higher than those in the survivor group. Unfortunately, the retrospective nature of this study prohibited reporting on the serum trajectories during CRRT. In several prior studies, patients with dysnatremia, including hyponatremia and hypernatremia, and sodium trajectories have been identified as at a higher risk of mortality than those in stable condition (Huang et al., 2024; Yessayan et al., 2021). Based on the above, many studies will be needed to examine the impact of dysnatremia and changes in sodium levels on CRRT outcomes.

In this multivariable Cox regression analysis, no association was found between BMI and elevated risk of 30-day in-hospital mortality in either group. This finding is inconsistent with previous research (H.-J. Lee & Son, 2020; Peters et al., 2023). This study included only a single BMI reading at baseline and did not account for changes in BMI over time. Thus, future studies on this issue should consider the body composition trajectory.

Based on the findings, comprehensive risk assessments should be conducted during CRRT. These findings may help health care providers develop strategies to reduce the risk of 30-day in-hospital mortality in critically ill patients on CRRT. Critical care nurses should be aware of the importance of identifying comorbid hepatic failure, early detection of deterioration in mental status and disease severity, and close monitoring of sodium levels during CRRT support to prevent avoidable adverse events in patients on CRRT.

### Limitations

This study was affected by a number of limitations. The relatively small sample size advises careful consideration when generalizing the findings to other populations. Multicenter, prospective cohort studies will be required to confirm our findings. Second, only patients on CRRT for more than 24 hours were included, which may lead to selection bias. Third, patients’ medical records may be missing in the process of collecting medical records, which may result in information bias. Also, indications for CRRT initiation were not included in this study. Finally, nutritional status, such as tube feeding, was not reflected in the analysis, making it impossible to demonstrate a relationship between nutritional support and mortality.

### Conclusions

Over half of the ICU patients undergoing CRRT experienced significantly higher 30-day in-hospital mortality. Based on the findings, ICU nurses should be more concerned about the impact of comorbid hepatic failure on adverse outcomes in patients requiring CRRT. In addition, the primary results emphasize the importance of closely observing patients’ consciousness level, illness severity score, and serum sodium levels after initiating CRRT. Significantly, early detection and management of these risk factors should be performed within 9 days of CRRT initiation. However, the pre- and intra-CRRT characteristics were not found to relate significantly to the risk of 30-day in-hospital mortality. Therefore, more studies are needed to investigate the comprehensive risk factors of in-hospital mortality among patients on CRRT using multicenter, larger cohort samples.

## References

[R1] Ahmadi S. Sarveazad A. Babahajian A. Ahmadzadeh K. Yousefifard M. 2023. Comparison of Glasgow Coma Scale and Full Outline of UnResponsiveness score for prediction of in-hospital mortality in traumatic brain injury patients: A systematic review and meta-analysis. European Journal of Trauma and Emergency Surgery, 49(4), 1693–1706. 10.1007/s00068-022-02111-w 36152069

[R2] Ahmed A. R. Obilana A. Lappin D. 2019. Renal replacement therapy in the critical care setting. Critical Care Research and Practice, 2019, Article ID 6948710. 10.1155/2019/6948710 PMC6664494 31396416

[R3] Bahtouee M. Eghbali S. S. Maleki N. Rastgou V. Motamed N. 2019. Acute physiology and chronic health evaluation II score for the assessment of mortality prediction in the intensive care unit: A single-centre study from Iran. Nursing in Critical Care, 24, 375–380. 10.1111/nicc.12401 30924584

[R4] Bodien Y. G. Barra A. Temkin N. R. Barber J. Foreman B. Vassar M. Robertson C. Taylor S. R. Markowitz A. J. Manley G. T. Giacino J. T. Edlow B. L. TRACK-TBI Investigators . 2021. Diagnosing level of consciousness: The limits of the Glasgow Coma Scale total score. Journal of Neurotrauma, 38(23), 3295–3305. 10.1089/neu.2021.0199 34605668 PMC8917895

[R5] Bourbonnais F. F. Slivar S. Malone-Tucker S. 2020. Caring for patients on CRRT—Key safety concerns identified by nurses. The Canadian Journal of Critical Care Nursing, 31(2), 13–19.

[R6] Chander S. Luhana S. Sadarat F. Parkash O. Rahaman Z. Wang H. Y. Kiran F. Lohana A. C. Sapna F. Kumari R. 2024. Mortality and mode of dialysis: Meta-analysis and systematic review. BMC Nephrology, 25, Article No. 1. 10.1186/s12882-023-03435-4 PMC10763097 38172835

[R7] Chang C.-H. Fan P.-C. Chang M.-Y. Tian Y.-C. Hung C.-C. Fang J.-T. Yang C.-W. Chen Y.-C. 2014. Acute kidney injury enhances outcome prediction ability of sequential organ failure assessment score in critically ill patients. PLOS ONE, 9(10), Article e109649. 10.1371/journal.pone.0109649 25279844 PMC4184902

[R8] Faul F. Erdfelder E. Buchner A. Lang A. G. 2009. Statistical power analyses using G*Power 3.1: Tests for correlation and regression analyses. Behavior Research Methods, 41(4), 1149–1160. 10.3758/BRM.41.4.1149 19897823

[R9] Fitzgerald M. Tan T. Rosenfeld J. V. Noonan M. Tee J. Ng E. Mathew J. Broderick S. Kim Y. Groombridge C. Udy A. Mitra B. 2022. An initial Glasgow Coma Scale score of 8 or less does not define severe brain injury. Emergency Medicine Australasia, 34(3), 459–461. 10.1111/1742-6723.13937 35220682 PMC9303457

[R10] Gong Y. Ding F. Zhang F. Gu Y. 2019. Investigate predictive capacity of in-hospital mortality of four severity score systems on critically ill patients with acute kidney injury. Journal of Investigative Medicine, 67(8), 1103–1109. 10.1136/jim-2019-001003 31575668 PMC6900215

[R11] Hadem J. Kielstein J. T. Manns M. P. Kümpers P. Lukasz A. 2019. Outcomes of renal dysfunction in patients with acute liver failure. United European Gastroenterology journal, 7, 388–396. 10.1177/2050640618817061 31019707 PMC6466757

[R12] Han S. S. Bae E. Kim D. K. Kim Y. S. Han J. S. Joo K. W. 2016. Dysnatremia, its correction, and mortality in patients undergoing continuous renal replacement therapy: A prospective observational study. BMC Nephrology, 17, Article No. 2. 10.1186/s12882-015-0215-1 26732402 PMC4702339

[R13] Huang S. Li X. Chen B. Zhong Y. Li Y. Huang T. 2024. Association between serum sodium trajectory and mortality in patients with acute kidney injury: A retrospective cohort study. BMC Nephrology, 25, Article No. 152. 10.1186/s12882-024-03586-y 38698368 PMC11067220

[R14] Husain-Syed F. Takeuchi T. Neyra J. A. Ramírez-Guerrero G. Rosner M. H. Ronco C. Tolwani A. J. 2023. Acute kidney injury in neurocritical care. Critical Care, 27(1), Article No. 341. 10.1186/s13054-023-04632-1 37661277 PMC10475203

[R15] Järvisalo M. J. Kartiosuo N. Hellman T. Uusalo P. 2022. Predicting mortality in critically ill patients requiring renal replacement therapy for acute kidney injury in a retrospective single-center study of two cohorts. Scientific Reports, 12(1), Article No. 10177. 10.1038/s41598-022-14497-z 35715577 PMC9205979

[R16] Jeon Y. H. Kim I. Y. Jang G. S. Song S. H. Seong E. Y. Lee D. W. Lee S. B. Kim H. J. 2021. Clinical outcomes and prognostic factors of mortality in liver cirrhosis patients on continuous renal replacement therapy in two tertiary hospitals in Korea. Kidney Research and Clinical Practice, 40(4), 687–697. 10.23876/j.krcp.21.033 34510860 PMC8685364

[R17] Jiang Y. Chen J. Yu Y. Yang F. Hamza M. Zou P. Wen A. Wu H. Zhang Y. 2022. Risk factors for the in-hospital mortality of CRRT-therapy patients with cardiac surgery-associated AKI: A single-center clinical study in China. Clinical and Experimental Nephrology, 26(12), 1233–1239. 10.1007/s10157-022-02274-1 36083528 PMC9668795

[R18] Kee Y. K. Kim D. Kim S. J. Kang D. H. Choi K. B. Oh H. J. Ryu D. R. 2018. Factors associated with early mortality in critically ill patients following the initiation of continuous renal replacement therapy. Journal of Clinical Medicine, 7(10), Article 334. 10.3390/jcm7100334 30297660 PMC6210947

[R19] Lee H.-J. Son Y.-J. 2020. Factors associated with in-hospital mortality after continuous renal replacement therapy for critically ill patients: A systematic review and meta-analysis. International Journal of Environmental Research and Public Health, 17(23), Article 8781. 10.3390/ijerph17238781 33256008 PMC7730748

[R20] Lee K. Y. Park K. Lee S. Jang J. Y. Bae K. S. 2022. Risk factors associated with 30-day mortality in patients with postoperative acute kidney injury who underwent continuous renal replacement therapy in the intensive care unit. Journal of Acute Care Surgery, 12(2), 47–52. 10.17479/jacs.2022.12.2.47

[R21] Mistry K. 2019. Dialysis disequilibrium syndrome prevention and management. International Journal of Nephrology and Renovascular Disease, 12, 69–77. 10.2147/IJNRD.S165925 31118737 PMC6503314

[R22] Nishino T. Kubota Y. Kashiwagi T. Hirama A. Asai K. Yasutake M. Kumita S. 2024. Hepatic function markers as prognostic factors in patients with acute kidney injury undergoing continuous renal replacement therapy. Renal Failure, 46(1), Article 2352127. 10.1080/0886022X.2024.2352127 38771116 PMC11110873

[R23] Park H. Yang J. Chun B. C. 2023. Assessment of severity scoring systems for predicting mortality in critically ill patients receiving continuous renal replacement therapy. PLOS ONE, 18(5), Article e0286246. 10.1371/journal.pone.0286246 37228073 PMC10212150

[R24] Park S. Lee S. Jo H. A. Han K. Kim Y. An J. N. Joo K. W. Lim C. S. Kim Y. S. Kim H. Kim D. K. 2018. Epidemiology of continuous renal replacement therapy in Korea: Results from the National Health Insurance Service claims database from 2005 to 2016. Kidney Research and Clinical Practice, 37(2), 119–129. 10.23876/j.krcp.2018.37.2.119 29971207 PMC6027810

[R25] Peters B. J. Barreto E. F. Mara K. C. Kashani K. B. 2023. Continuous renal replacement therapy and mortality in critically ill obese adults. Critical Care Explorations, 5(11), Article e0998. 10.1097/CCE.0000000000000998 38304705 PMC10833633

[R26] Petnak T. Thongprayoon C. Cheungpasitporn W. Shawwa K. Mao M. A. Kashani K. B. 2022. The prognostic importance of serum sodium for mortality among critically ill patients requiring continuous renal replacement therapy. Nephron, 146(2), 153–159. 10.1159/000519686 34794149

[R27] Prasad B. Urbanski M. Ferguson T. W. Karreman E. Tangri N. 2016. Early mortality on continuous renal replacement therapy (CRRT): The prairie CRRT study. Canadian Journal of Kidney Health and Disease, 3, Article 36. 10.1186/s40697-016-0124-7 PMC4957309 27453787

[R28] Priyamvada P. S. Jayasurya R. Shankar V. Parameswaran S. 2018. Epidemiology and outcomes of acute kidney injury in critically ill: Experience from a tertiary care center. Indian Journal of Nephrology, 28(6), 413–420. 10.4103/ijn.IJN_191_17 30647494 PMC6309393

[R29] Reith F. C. Van den Brande R. Synnot A. Gruen R. Maas A. I. 2016. The reliability of the Glasgow Coma Scale: A systematic review. Intensive Care Medicine, 42(1), 3–15. 10.1007/s00134-015-4124-3 26564211

[R30] Rewa O. G. Ortiz-Soriano V. Lambert J. Kabir S. Heung M. House A. A. Monga D. Juncos L. A. Secic M. Piazza R. Goldstein S. L. Bagshaw S. M. Neyra J. A. 2023. Epidemiology and outcomes of AKI treated with continuous kidney replacement therapy: The multicenter CRRTnet study. Kidney Medicine, 5(6), Article 100641. 10.1016/j.xkme.2023.100641 37274539 PMC10238597

[R31] Schaffer P. Chowdhury R. Jordan K. DeWitt J. Elliott J. Schroeder K. 2022. Outcomes of continuous renal replacement therapy in a community health system. Journal of Intensive Care Medicine, 37(8), 1043–1048. 10.1177/08850666211052871 34812078

[R32] Sharma B. Bhateja A. Sharma R. Chauhan A. Bodh V. 2024. Acute kidney injury in acute liver failure: A narrative review. Indian Journal of Gastroenterology, 43(2), 377–386. 10.1007/s12664-024-01559-5 38578564

[R33] Tandukar S. Palevsky P. M. 2019. Continuous renal replacement therapy: Who, when, why, and how. Chest, 155(3), 626–638. 10.1016/j.chest.2018.09.004 30266628 PMC6435902

[R34] Tiglis M. Peride I. Florea I. A. Niculae A. Petcu L. C. Neagu T. P. Checherita I. A. Grintescu I. M. 2022. Overview of renal replacement therapy use in a general intensive care unit. International Journal of Environmental Research and Public Health, 19(4), Article 2453. 10.3390/ijerph19042453 35206640 PMC8878091

[R35] Tujios S. R. Hynan L. S. Vazquez M. A. Larson A. M. Seremba E. Sanders C. M. Lee W. M. Acute Liver Failure Study Group. 2015. Risk factors and outcomes of acute kidney injury in patients with acute liver failure. Clinical Gastroenterology and Hepatology, 13(2), 352–359. 10.1016/j.cgh.2014.07.011 25019700 PMC4937794

[R36] Uusalo P. Hellman T. Järvisalo M. J. 2021. Mortality and associated risk factors in perioperative acute kidney injury treated with continuous renal replacement therapy. Perioperative Medicine, 10, Article No. 57. 10.1186/s13741-021-00227-y 34903294 PMC8670067

[R37] Wang H. Kang X. Shi Y. Bai Z. H. Lv J. H. Sun J. L. Pei H. H. 2020. SOFA score is superior to APACHE-II score in predicting the prognosis of critically ill patients with acute kidney injury undergoing continuous renal replacement therapy. Renal Failure, 42(1), 638–645. 10.1080/0886022X.2020.1788581 32660294 PMC7470067

[R38] Xia Z.-J. He L.-Y. Pan S.-Y. Cheng R.-J. Zhang Q.-P. Liu Y. 2021. Disease severity determines timing of initiating continuous renal replacement therapies: A systematic review and meta-analysis. Frontiers in Medicine, 8, Article 580144. 10.3389/fmed.2021.580144 34869398 PMC8636750

[R39] Yang S. Wang Z. Liu Z. Wang J. Ma L. 2016. Association between time of discharge from ICU and hospital mortality: A systematic review and meta-analysis. Critical Care, 20(1), Article 390. 10.1186/s13054-016-1569-x 27903270 PMC5131545

[R40] Yessayan L. T. Szamosfalvi B. Rosner M. H. 2021. Management of dysnatremias with continuous renal replacement therapy. Seminars in Dialysis, 34(6), 472–479. 10.1111/sdi.12983 34218456

[R41] Zhou Z. Liu C. Yang Y. Wang F. Zhang L. Fu P. 2023. Anticoagulation options for continuous renal replacement therapy in critically ill patients: A systematic review and network meta-analysis of randomized controlled trials. Critical Care, 27(1), Article No. 222. 10.1186/s13054-023-04519-1 37287084 PMC10249230

